# Outcome measurement in trauma-informed care education across healthcare settings: A scoping review

**DOI:** 10.1017/gmh.2026.10277

**Published:** 2026-07-23

**Authors:** Analpa Vishwanath, Louloua Ashikhusein Waliji, Abdullah Bengali, Sabina Rajkumar, Negar Sayrefidazeh, Sophie Soklaridis, Dana C. Ross

**Affiliations:** 1 McMaster University Faculty of Health Sciences, Canada; 2 Women’s College Hospital, Canada; 3 University of Toronto, Canada; 4 CAMH, Canada

**Keywords:** implementation, outcome measurement, healthcare provider, trauma, multidisciplinary providers

## Abstract

As trauma-informed care (TIC) becomes more integrated into healthcare systems, consistent and meaningful evaluation remains limited. Educational interventions are central to implementation, yet outcome measures vary widely. This scoping review systematically mapped outcome measures used to evaluate TIC educational interventions in healthcare settings to identify current practices, methodological gaps and opportunities for more coherent and context-sensitive assessment. The review followed PRISMA-ScR guidelines. Five databases (MEDLINE, EMBASE, PsycINFO, CINAHL, ERIC) were searched from database inception to October 2024. Eligible studies described TIC educational interventions for healthcare providers and reported at least one outcome related to knowledge, attitudes, confidence, competence, or behavior. Two reviewers independently screened and extracted data on study design, participants, intervention characteristics and outcome measures. Outcomes were categorized using Moore’s Expanded Outcomes Framework, which includes seven hierarchical levels from participation to patient or community health outcomes. Twenty-eight studies met inclusion criteria, most published after 2020 and conducted in the United States. Outcome measurement was dominated by self-reported data, primarily custom pre–post surveys assessing knowledge, confidence or attitudes. Only eight studies used validated tools, most commonly the ARTIC-35 or ARTIC-45. Few assessed behavioral or system-level change, and none directly measured patient outcomes. Educational formats ranged from brief online modules to multi-session interdisciplinary trainings. A small subset explored relational or equity-oriented outcomes such as empathy or cultural humility, often using qualitative methods. This review highlights the field’s reliance on short-term, self-reported outcomes and limited use of validated or standardized measures. Developing accessible, equity-informed tools that capture behavioral and system-level change is essential to advance research, guide policy and sustain trauma-informed transformation across healthcare systems.

## Impact statement

Trauma-informed care (TIC) is increasingly recognized as a cornerstone of equitable and compassionate healthcare, yet there remains little consensus on how to evaluate its impact in education and practice. To our knowledge, this is the first review to systematically map the outcome measures used to assess TIC educational interventions across healthcare settings. Using Moore’s Expanded Outcomes Framework, the review provides a structured synthesis of how learning, behavioral and system-level outcomes have been defined and measured to date.

Findings reveal an overreliance on short-term, self-reported indicators of knowledge and confidence, with limited use of validated or equity-informed tools. Few studies examined sustained changes in provider behavior or organizational transformation, and almost none evaluated patient or community outcomes. These gaps hinder comparability across interventions and limit the ability to assess long-term impact or justify investment in large-scale implementation.

By identifying key methodological and conceptual gaps, this review underscores the need for standardized, accessible and context-sensitive outcome measures that reflect the relational and systemic dimensions of TIC. The findings can inform future research design, funding priorities and policy decisions aimed at embedding trauma-informed principles across health systems. The Supplementary Tables accompanying this review provide a practical reference for educators, evaluators and implementation scientists seeking to strengthen the rigor and relevance of TIC evaluation.

## Introduction

Trauma is a major global public health concern that affects individuals, communities and health systems worldwide. Recent meta-analyses estimate that posttraumatic stress disorder (PTSD) affects about 2% of adults in economically developed regions and up to 16% in conflict-affected or less-resourced settings, while a global review found an overall prevalence of 6.2% for complex PTSD, with higher rates in clinical and high-risk groups (Fung et al., 2025; Huynh et al., 2025). Exposure to trauma is associated with elevated risks of chronic disease, mental illness, substance use and premature mortality (Senaratne et al., 2024; Thurston et al., 2025). The cumulative burden of trauma contributes to health disparities and places additional pressure on already strained health systems through higher service use and long-term care costs (Al Jowf et al., 2022; Davis et al., 2022). Global frameworks such as the WHO QualityRights initiative and national mental-health strategies increasingly emphasize trauma-informed and equity-oriented approaches as essential dimensions of quality care and workforce well-being (WHO, 2021; PHAC, 2022).In response, trauma-informed care (TIC) has emerged as an organizational and systems-level framework used across sectors such as healthcare, education, social services and justice systems to integrate awareness of trauma’s impact into service delivery and organizational culture (Goldstein et al., 2024; Yildiz et al., 2024). Within healthcare specifically, TIC aims to enhance safety, trust and healing by embedding trauma awareness into interactions, practices and systems that shape care experiences for both patients and providers (SAMHSA, 2014). Evaluating outcomes in healthcare-based TIC education is therefore critical to ensure that training efforts translate into lasting improvements in care quality and health equity.

Trauma can result from experiences such as abuse, neglect, violence, systemic inequity or chronic stress, and its effects often extend across physical, psychological and relational domains. In healthcare settings, these experiences can shape how people perceive safety, interpret interactions and engage with providers. TIC recognizes these dynamics and seeks to ensure that all aspects of care are delivered with an understanding of trauma’s potential impact. Its core principles include safety, trustworthiness, collaboration, peer support, empowerment and attention to cultural, historical and gender factors (SAMHSA, 2014). These principles guide organizations toward practices that reduce harm and promote safety for both patients and staff. Implementing TIC therefore represents a structural shift in healthcare, embedding trauma awareness into policies, practices and everyday interactions to create environments that support recovery and resilience (Huo et al., 2023; McBain and Cordova, 2024). These developments reflect a broader movement toward relationship-centered and equity-oriented models that link clinical safety with organizational culture and staff well-being (Waite and Nardi, 2024; Ross et al., 2025).

Education and training are central to translating TIC principles into everyday practice, yet their scope, intensity and delivery vary widely across healthcare (Berring et al., 2024). Some initiatives are short awareness sessions designed to introduce key concepts, while others are multi-component programs embedded within professional curricula, quality-improvement initiatives or broader organizational change strategies (Burns et al., 2023). Training has been implemented in acute and primary care, mental health, pediatrics, long-term care and community-based services, each shaped by distinct clinical priorities, resource constraints and workforce cultures (Chin et al., 2024; Stokes et al., 2024). This variability highlights the adaptability of TIC to different care contexts but also exposes tension between scalability and depth. Programs designed for rapid uptake may sacrifice relational or reflective components, while more comprehensive approaches often require organizational commitment and leadership engagement to sustain change (McBain and Cordova, 2024; Simons et al., 2024). These differences make it difficult to compare effectiveness or to identify core educational ingredients that drive change across settings. As a result, evidence on what constitutes effective TIC education remains fragmented, underscoring the need for shared frameworks that link implementation strategies with measurable outcomes (Han et al., 2021; Mahon, 2024).

Evaluation of TIC education remains inconsistent across the literature. Many studies rely on self-reported outcomes such as satisfaction, confidence or attitudes toward TIC (Stillerman et al., 2023), which offer insight into feasibility and short-term learning but reveal little about sustained behavior change, patient experience or system-level impact (Gelezelyte et al., 2023). Stillerman and colleagues further note that evaluation efforts rarely extend beyond immediate educational outcomes, leaving uncertainty about how TIC principles translate into organizational or clinical practice. As noted by Purtle (2020), most evaluations of trauma-informed organizational interventions rely on self-reported staff outcomes with limited follow-up or standardized measurement. A few validated instruments exist, including the Attitudes Related to Trauma-Informed Care (ARTIC) scales, yet their use is limited and often modified to fit specific settings or populations (Champine et al., 2019; Mendez et al., 2023). Given the limited availability of validated tools, many studies have developed their own context-specific measures to assess knowledge, competence or perceived safety, resulting in conceptual and methodological diversity across the field (King et al., 2019; Hanson et al., 2024). Few evaluations incorporate multi-method or longitudinal designs capable of capturing behavioral change or organizational outcomes over time. While several reviews have examined TIC implementation and organizational change (Goldstein et al., 2024; Nguyen-Feng et al., 2025), few have focused specifically on how outcomes are defined and measured within educational interventions. Consequently, the field lacks consensus on what meaningful change looks like in TIC education or how to assess it across diverse healthcare disciplines and contexts.

Despite growing international adoption of TIC frameworks and training initiatives, there has been no systematic synthesis of how outcomes are defined and measured within educational interventions for healthcare providers. Recent curricular analyses further underscore this gap, noting that evaluation approaches remain limited and inconsistently applied (Gerber et al., 2024). Recent reviews have advanced understanding of TIC implementation across settings and populations but have not examined the educational domain in depth or the tools used to evaluate learning outcomes (Berring et al., 2024; Bell et al., 2025). Consistent, theory-driven evaluation is essential to move the field beyond descriptive reporting toward evidence-informed improvement. Although this review focuses on educational interventions for healthcare providers, TIC education is often conceptualized as a foundational strategy for broader implementation and organizational change within healthcare systems. As a result, outcome evaluation in TIC education may extend beyond immediate learning outcomes to include behavioral, relational and systems-level impacts. To provide structure and enable comparison across diverse studies, this review applied Moore’s Expanded Outcomes Framework (Moore et al., 2009), a model widely used to evaluate educational interventions across multiple levels of learning and practice. Using this framework, the review maps how outcomes have been defined, measured and reported in TIC educational interventions for healthcare providers, identifying methodological gaps and opportunities for improvement across levels of learning and system impact.

## Methods

### Review design and research question

This scoping review followed the Preferred Reporting Items for Systematic Reviews and Meta-Analyses Extension for Scoping Reviews (PRISMA-ScR) guidelines (Tricco et al., 2018). The protocol for this review was registered on the Open Science Framework (OSF) (Foster and Deardorff, 2017) at https://doi.org/10.17605/OSF.IO/AG572.

The research question guiding this review was: How are outcomes defined, measured and reported in educational interventions on TIC for healthcare providers? The review question, eligibility criteria and search strategy were developed using the Population–Concept–Context (PCC) framework recommended by the Joanna Briggs Institute for scoping reviews (Peters et al., 2020). The Population comprised healthcare providers and healthcare trainees. The Concept focused on TIC educational interventions and the outcome measures used to evaluate them. The Context included healthcare-related educational and clinical settings across diverse healthcare systems. These PCC elements informed the development of the review question, inclusion criteria and search strategy.

To support consistent categorization of outcomes during data synthesis, Moore’s Expanded Outcomes Framework (Moore et al., 2009) was applied. Originally developed to evaluate continuing medical education, the framework includes seven hierarchical levels ranging from participation and satisfaction to knowledge, competence, performance and patient- or community-level health outcomes.

### Search strategy

A comprehensive, multi-stage search strategy was developed in collaboration with an experienced research librarian. An initial exploratory search was conducted in MEDLINE and Google Scholar to identify relevant terminology related to TIC and healthcare education. These terms were used to inform a broader strategy that was applied to five electronic databases: MEDLINE, EMBASE, APA PsycInfo, CINAHL and ERIC. The final search strategy combined controlled vocabulary and keywords relating to TIC, education and training, healthcare professionals and outcome measurement. The search was conducted from the inception of each included database to October 23, 2024. Full search strategies for each database are available in Supplementary File S1. Reference lists of all included studies were also screened to identify additional eligible literature. Studies available electronically ahead of print and indexed in the searched databases before October 23, 2024 were eligible for inclusion, even if they were subsequently assigned to a 2025 print issue.

### Eligibility criteria and study selection

This review focused on studies involving healthcare providers, including but not limited to physicians, nurses, allied health professionals, health administrators and students in health professions. Studies were included if they described an educational intervention specifically designed to improve the delivery of TIC and reported at least one outcome related to knowledge retention, attitudes toward TIC, competencies in providing TIC, provider confidence and the usability of the intervention. Interventions could be delivered in any healthcare setting, including hospitals, primary care, long-term care, mental-health centers or pediatric care settings. Educational interventions were considered eligible if they explicitly identified TIC or closely related terminology (e.g., trauma-aware or trauma-sensitive approaches), as the primary focus of the training. Studies were eligible if they used qualitative, quantitative or mixed-method designs and were published in peer-reviewed journals.

Studies were excluded if they were review articles, protocols, conference abstracts without full papers, book chapters, unpublished studies or gray literature. Gray literature was excluded to focus on validated outcome measures and more rigorously evaluated interventions, as well as to ensure feasibility given time and resource constraints. Studies published in languages other than English were also excluded. In addition, studies were ineligible if they described educational interventions that did not include outcome data, or if the intervention was trauma-informed in delivery but not focused on TIC as its subject.

Search results were uploaded to Covidence systematic review software, where duplicates were removed. Titles and abstracts were screened by two independent reviewers from a team of three. Full texts were obtained for all potentially relevant studies and screened using the same criteria. Discrepancies at any stage were resolved with the input of a third reviewer. The study selection process and reasons for exclusion at the full-text stage are presented in the PRISMA flow diagram.

For this review, ‘Context’ referred to healthcare-related educational and clinical settings in which TIC educational interventions were implemented, including hospitals, primary care, mental health services, pediatric care and other healthcare delivery environments.

### Data analysis

Data were extracted using a structured form that was developed and pilot-tested by the review team. For each study, reviewers recorded information on study design, setting, country, sample size, professional roles of participants and healthcare context. Intervention characteristics were also extracted, including format, duration, delivery method and stated objectives. The outcome measures reported in each study were documented along with the tools or instruments used, where applicable.

Particular attention was given to outcomes such as knowledge acquisition, changes in provider attitudes or confidence, perceived or demonstrated competence, changes in clinical practice and any reported patient- or system-level effects. Outcomes were categorized using Moore’s Expanded Outcomes Framework, which allowed for comparison of the types and levels of outcomes being measured across studies. This framework includes seven levels: participation, satisfaction, declarative knowledge, procedural knowledge, competence, performance and health outcomes at the patient or community level. In addition to mapping outcomes to Moore’s Expanded Outcomes Framework, the review team also documented whether studies reported outcomes related to relational practice or equity-informed processes, such as empathy, cultural humility or attention to emotional safety. These outcomes were identified through both direct measurement and qualitative descriptions within the included studies.

Data extraction was conducted independently by two reviewers from a team of three. Discrepancies were discussed until consensus was reached. All extracted data were managed within Covidence. Findings were synthesized through narrative analysis to highlight trends, common approaches and gaps in outcome measurement practices across included studies.

## Results

### Study characteristics

**Figure 1.**
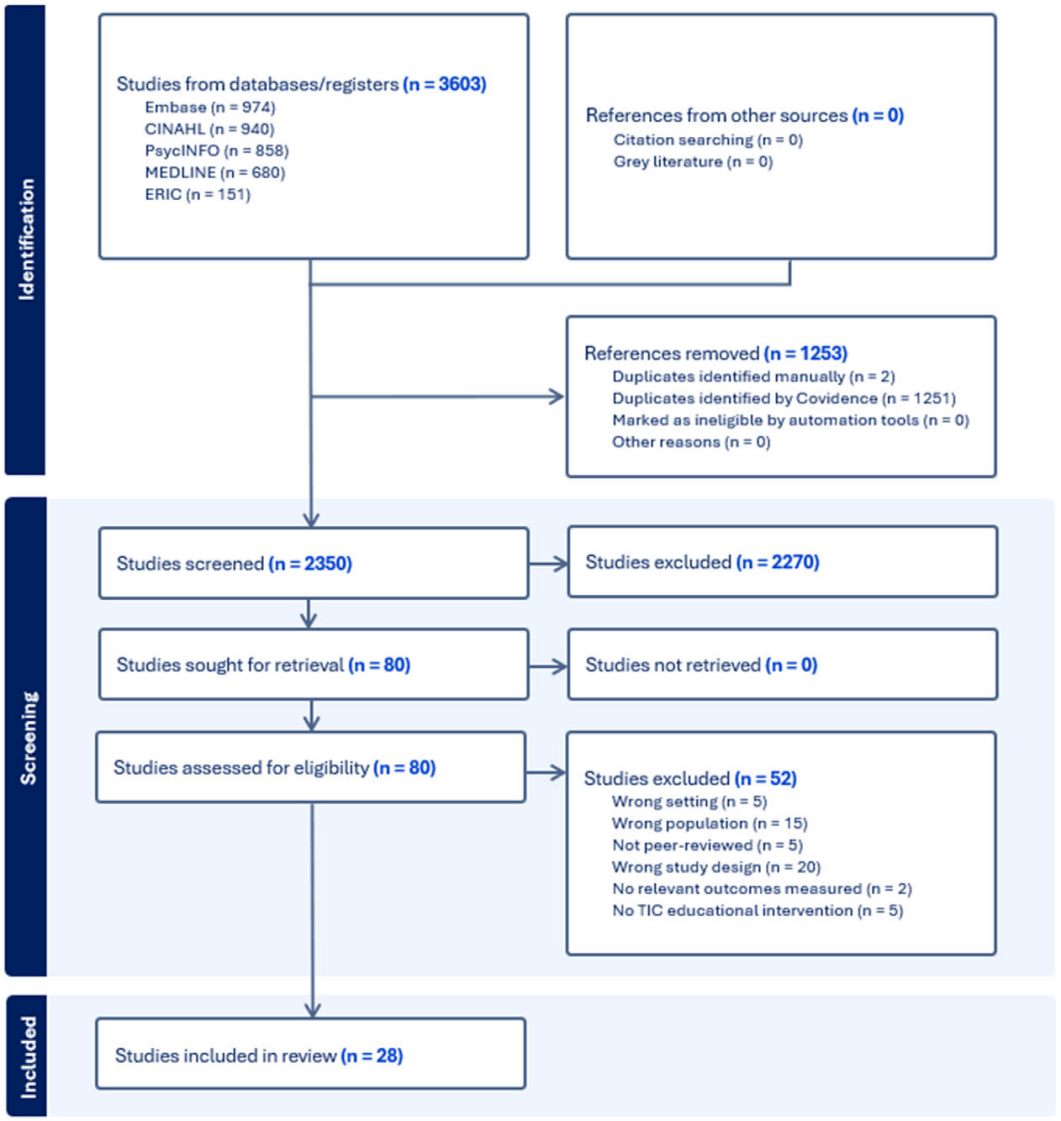
PRISMA flow diagram of study inclusion. Figure 1. long description.

A total of 28 studies met the inclusion criteria for this review. The study selection process is presented in the PRISMA-ScR flow diagram (Figure 1), and study characteristics are summarized in Table 1.

**Table 1.** Study characteristic Table 1. long description.

Author & year of publication	Country	Study design	Setting	Participants	Duration of intervention	Intervention	Primary measure
Brown et al. (2021)	United States	Quasi-experimental pre-test post-test	Medical school and hospital	First-year medical and dental students	3.5 h	In-person session with didactics, demo, discussion, role-plays	Custom pre/post surveys (0–100 scales; 5-point Likert scale)
Raja et al. (2015)	United States	Program evaluation study	Dental school	Second-year dental students	Two 3.5-h sessions	In-person lectures with videos, role-play, mock chart notes	Custom pre/post multiple-choice test, chart note evaluation
Weiss et al. (2017)	United States	Quasi-experimental pre-test post-test	Large pediatric healthcare network	Mixed clinical and admin staff	1-h workshop	In-person workshop with didactics, discussion, case examples	Custom Trauma-Informed Medical Care Questionnaire
McEvedy et al. (2017)	Australia	Descriptive qualitative study	Public mental health services	Multidisciplinary mental health staff	Two days	In-person train-the-trainer program with didactics, activities and vignettes	Custom semi-structured interviews, focus groups, paired in-depth interviews
Schmitz et al. (2019)	United States	Quasi-experimental pre-test post-test	Pediatric residency program	Pediatric residents	25 min	Online self-paced module	Custom pre/post surveys (5-point Likert scale)
Niimura et al. (2019)	Japan	Quasi-experimental pre-test post-test	Psychiatric hospitals	Mental health professionals	4.5 h	In-person lecture and group discussion	ARTIC-45 (pre-post); ARTIC-35 (follow-up)
Shamaskin-Garroway et al. (2020)	United States	Program evaluation study	VA primary care residency program	Physician and nurse practitioner residents	1 academic year	In-person didactics, reflection rounds, observed encounters	Custom self-report questionnaire (CVI-validated)
Randall et al. (2020)	United States	Quasi-experimental pre-test post-test	Midwestern U.S. university (PT/OT program)	Second-year PT and OT students	4 h	In-person simulation-based training	Custom Likert scale; General Self-Efficacy Scale; Adult Hope Scale
Chokshi et al. (2020)	United States	Quasi-experimental pre-test post-test	Children’s National Hospital	Pediatric residents, physicians, medical learners	2 h	Online self-paced e-modules	Custom 17-item questionnaire (5-point Likert scale)
Chokshi et al. (2020)	United States	Program evaluation study	School of Medicine and Health Sciences	Second-year medical students	4-h symposium	In-person didactics, small-group case discussion	Custom post-survey (5-point Likert scale; open-ended items)
Dublin et al. (2021)	United States	Mixed methods research	Mental health organizations	Mental health professionals	Varied (~4–8 h)	In-person case-based and problem-based learning	Custom follow-up survey (5-point scale; qualitative responses)
Gore et al. (2021)	United States	Quasi-experimental pre-test post-test	Rush Medical College	Second-year medical students	3 h	In-person lecture and small group activities	Custom pre/post survey (5-point Likert scale)
Chokshi and Goldman (2021)	United States	Mixed methods research	George Washington University Hospital	Internal medicine residents (PGY1–3)	4 h	Online didactics, breakout rooms, SP video, case-based discussion	Custom pre/post survey (5-point Likert scale)
Berg-Poppe et al. (2022)	United States	Quasi-experimental pre-test post-test	Pediatric clinics, schools, inpatient, community healthcare settings	Interprofessional pediatric service providers	4 h plus prereading	In-person interactive session (activities, case discussion, guided dialogue)	Custom pre/post survey (16-item Likert scale)
(Cerny et al., 2023)	United States	Mixed methods research	Interprofessional continuing education program	Pediatric healthcare professionals	6-week self-study, 4-h in-person	Hybrid: online modules, readings, in-person case discussion	ARTIC-35 (pre/post)
Brennan et al. (2023)	United States	Quasi-experimental pre-test post-test	Rush Medical College	Fourth-year medical students	2 h	Online didactics and interactive clinical vignette, large group discussion	Custom pre/post (5-point Likert scale)
Buxton et al. (2023)	United States	Quasi-experimental pre-test post-test	Surgical residency program	Surgical interns	4 h (over 2 sessions)	In-person, peer-to-peer	Custom pre/post (5-point Likert scale)
Ford et al. (2021)	United States	Quality improvement	NICU and special care nurseries	Multidisciplinary NICU and nursery staff	2.5 years	Hybrid (in-person twice yearly meetings & monthly virtual sessions)	Modified NCETA Attitude Measurement: Brief Scales (5-point Likert scale)
Lee et al. (2023)	United States	Quasi-experimental pre-test post-test	Medical Center: Simulated emergency department	Third and fourth-year medical students	2.5-h session	In-person didactic and simulation cases with debriefs	Custom pre/post (5-point Likert scale, multiple-choice, free text)
Wholeben et al. (2023)	United States	Quasi-experimental pre-test post-test	Trauma center, community organization, nursing schools	ED staff, staff advocates, nursing students	1.5 to 2 h	In-person (post COVID-19) or virtual; discussion and case vignettes	ARTIC-35 (pre/post)
Long et al. (2024)	Australia	Quasi-experimental static group comparison	Regional maternity health service	Midwives	2 days	In-person lecture and facilitated discussion	ARTIC-35 (post)
Franchek-Roa et al. (2024)	United States	Mixed methods research	Residency programs	Medical residents	4 h	In-person (didactics, virtual tour, discussion, role-playing)	Custom retrospective pre/post survey
Davis et al. (2024)	United States	Mixed methods research	Level IV Neonatal Intensive Care Unit	Multi-disciplinary NICU team members	One year (32 x 1-h sessions)	Online live sessions and experiential learning activities	ARTIC-35 (pre/post)
Hall et al. (2024)	United States	Quasi-experimental pre-test post-test	Children’s National Hospital	Pediatric primary care faculty and staff	1 h	Online live (didactics, case-based discussion)	Custom pre/post (5-point Likert scale)
Heffernan et al. (2024)	Ireland	Qualitative descriptive design	Child and Adolescent Mental Health inpatient unit	Mixed clinical staff	6 x 1-h sessions	In-person weekly didactic sessions	Custom semi-structured interviews (focus group and 1:1 interview)
Stewart et al. (2024)	Canada	Mixed methods research	Community agencies	Multidisciplinary mental health professionals	At least 12 h plus two seminars	Hybrid (in-person and online) or online only; didactic, seminars, online modules	ARTIC-45 (pre/post)
Kopstick et al. (2025)	United States	Quasi-experimental pre-test post-test	Academic medical conference	Mixed healthcare providers and staff	90 min	In-person workshop (didactics, role-playing, self-reflection, group discussion)	Custom pre/post survey (4-point Likert scale)
Lamberson et al. (2025)	United States	Quasi-experimental pre-test post-test	Academic emergency department	ED staff, EMTs, mental health technicians	4 h	In-person (didactic, role play, discussion, activities)	Validated CPTS Trauma-Informed Care Provider Survey

#### Study setting and participants

Included studies were published between 2015 and 2024, with the majority (*n* = 19, 68%) appearing from 2021 onward. The majority were conducted in the United States, which accounted for 23 of the 28 included studies. The remaining studies were conducted in Australia (*n* = 2), Canada (*n* = 1), Ireland (*n* = 1) and Japan (*n* = 1), indicating comparatively limited representation from non-US healthcare systems. Study settings included academic institutions, hospitals, mental health organizations, pediatric clinics and community-based organizations. Participants represented a range of professional roles, including medical and dental students, nurse practitioners, emergency department staff, allied health professionals, physicians and multidisciplinary teams.

Allied health professionals, such as social workers, mental health specialists, occupational therapists and physiotherapists, were the most represented group (*n* = 11, 39%). Nurses or nursing students were included in eight studies, and both medical students and physicians participated in six studies each. Sample sizes varied widely, from fewer than 20 participants to large-scale studies involving over 1,000. Most studies had sample sizes of around 100.

Demographic information was reported in 17 of the included studies; however, the type and extent of information reported varied. Seven studies reported participants’ gender or sex, and samples were predominantly female (48%–100%). Definitions of gender and sex were often unclear, and a few studies used the terms interchangeably or included limited categories such as “other” or “prefer not to say.” Few studies reported participants’ years of experience, education level, or prior TIC training exposure.

#### Training intervention formats

Training formats varied across studies. Of the 24 studies that explicitly reported delivery format, 13 were conducted in-person, three were synchronously online, three used a virtual asynchronous format, three used hybrid models and one retrospective study reported variable formats. In terms of duration, training length ranged from a single 25-min session to a multi-year implementation. Excluding one outlier, a single 2.5-year collaborative study (Ford et al., 2021), the average training duration was approximately 5.6 h, with most sessions around 4 h.

Many interventions combined didactic instruction with case studies, standardized patients, role plays and small-group discussions. Learning objectives commonly addressed foundational principles of TIC, adverse childhood experiences (ACEs) and the neurobiology of trauma. Several programs also emphasized skill development, including screening for trauma, responding to disclosures, engaging in trauma-informed communication and increasing provider self-awareness. Overall, these findings highlight the considerable variability in how TIC education is structured, from brief awareness sessions to multi-component, skill-based curricula.

#### Outcome measurements and tools

The most reported outcomes were knowledge acquisition, attitudes toward TIC and confidence or comfort in applying trauma-informed approaches. Knowledge acquisition was generally defined as participants’ understanding of core trauma-related concepts and was most frequently assessed through custom pre–post self-report surveys using Likert scale items. A smaller number of studies used multiple-choice tests or written reflections. Confidence and comfort were typically measured as participants’ perceived ability and preparedness to apply trauma-informed principles in care settings. These outcomes were assessed through self-reported pre–post surveys, that incorporated Likert scale items. Attitudes were defined as participants’ beliefs or perspectives related to TIC and patient interactions. Most studies used custom Likert scales, although several employed validated tools such as the Attitudes Related to Trauma-Informed Care (ARTIC-35) or ARTIC-45 (Baker et al., 2021).

Of the 28 included studies, 20 used custom self-report surveys to assess primary outcomes. These often included pre–post Likert scales and open-ended qualitative questions. Eight studies used at least one validated, standardized measure: six used either the ARTIC-35 or ARTIC-45. One study employed the TIC Provider Assessment Survey (Hanson et al., 2024), while another used the General Self-Efficacy Scale (Schwarzer and Jerusalem, 1995) and the Adult Hope Scale (Snyder et al., 1991).

Two studies used qualitative methods, such as focus groups, interviews, or open-ended responses, as their primary approach to outcome assessment. An additional 12 studies incorporated qualitative methods to assess secondary outcomes, which included interprofessional collaboration, team climate, perceived barriers and facilitators to implementation, and leadership support for TIC. Timing of outcome measurement also varied. Most studies assessed outcomes immediately following the intervention, while 10 studies included at least one follow-up timepoint ranging from one to 24 months post-training. Most follow-ups occurred within 3 months of the intervention.

#### Moore’s expanded outcomes framework

**Figure 2.**
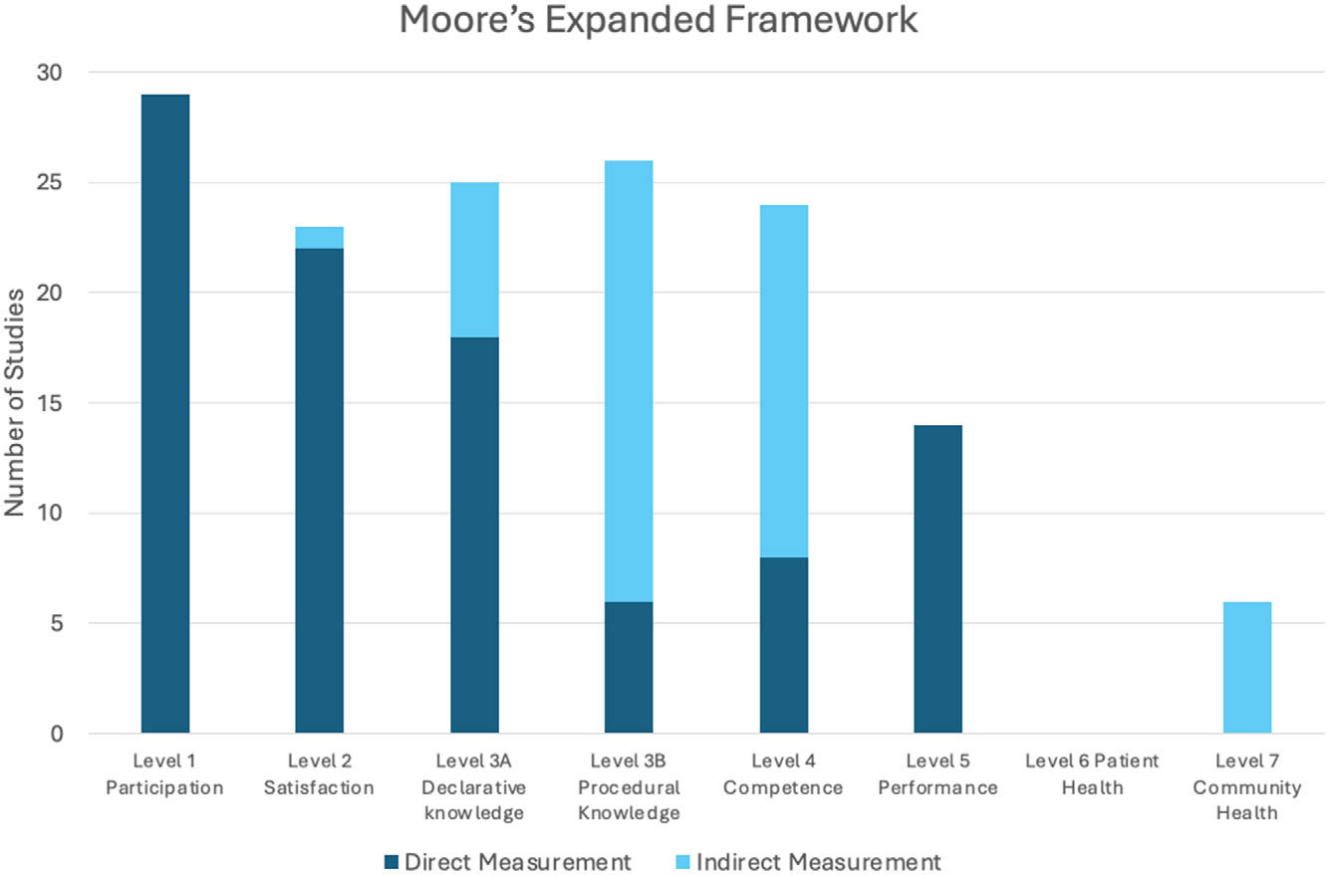
Distribution of outcome measures across Moore’s Expanded Outcomes Framework. Figure 2. long description.

Figure 2 illustrates how outcome measures were distributed across Moore’s Expanded Outcomes Framework. For each level, outcomes were categorized as either directly or indirectly measured, depending on whether data reflected explicit measurement of the target construct or inferred change through related indicators.

All included studies directly reported Level 1 (Participation), typically through attendance records or completion data. At Level 2 (Satisfaction), most studies used direct measurement through post-training surveys, while one study inferred satisfaction from high levels of participant engagement.

At Level 3A (Declarative Knowledge), direct measures included pre–post knowledge assessments or self-reported knowledge gains. Some studies inferred knowledge acquisition through tools like the ARTIC-35 or ARTIC-45, which primarily assess attitudes and were thus considered indirect indicators at this level. Level 3B (Procedural Knowledge) was more often assessed indirectly, inferred from engagement in skill-based activities such as case studies, role plays, or standardized patient encounters rather than from observed demonstrations.

While nearly all studies reported participation and satisfaction outcomes, very few assessed higher-level indicators of change. Only a small number examined Level 4 (Competence) or Level 5 (Performance) outcomes within Moore’s Expanded Outcomes Framework. Competence was typically assessed through self-reported ability to apply trauma-informed skills, and in one study, through observed performance during training. Indirect measures, such as self-reported confidence or stated intention to change clinical behavior, were more common and may reflect perceived rather than demonstrated competence. At the performance level, outcomes included reported changes to clinical practice or professional behaviors following training, but these were rarely corroborated by objective data. No studies evaluated outcomes at Level 6 (Patient Health), and just one captured indirect Level 7 (Community Health) indicators, such as organizational policy change, leadership endorsement or perceived improvements in system-level readiness for TIC. Together, these findings reveal a significant evidence gap: most evaluations focus on immediate learning rather than sustained behavioral or system-level impact.

#### Relational and equity-informed process outcomes

A small but growing number of studies are beginning to operationalize relational and equity-centered constructs as measurable outcomes, marking a shift in TIC education from focusing solely on individual learning to capturing how training may influence interpersonal dynamics, empathy and equity-oriented practice. Several studies explicitly measured outcomes in these domains, including bias awareness, cultural humility and empathy – concepts most often explored through qualitative methods such as open-text survey responses or focus groups. Others examined these outcomes indirectly using attitudinal scales or post-intervention interviews.

For example, Randall et al., (2020) used the Adult Hope Scale (Snyder et al., 1991) to assess provider resilience through the lens of hope. Dublin et al. (2021) evaluated long-term learning retention (6–24 months post-intervention) and its impact on clinical practice among mental-health professionals who completed the Core Curriculum on Childhood Trauma (CCCT); their survey combined Likert-scale items on trauma-related skills with open-ended questions, revealing increased empathy and heightened awareness of bias in clinical reasoning. Cerny et al. (2023) examined an interprofessional TIC curriculum for pediatric providers using structured focus groups and written reflections 2 months post-training, with participants describing greater empathy and a stronger sense of empowerment in their clinical work.

### Quality of studies

Consistent with scoping review methodology, a formal quality appraisal of included studies was not conducted. However, methodological features such as study design, sample characteristics and reporting transparency were documented to support interpretation of the findings.

## Discussion

### Overview

This scoping review mapped how outcomes have been defined, measured and reported in educational interventions on TIC for healthcare providers. Twenty-eight studies published between 2015 and 2024 were identified, most conducted in the United States across academic, hospital and community-based settings. Overall, evaluations of TIC education demonstrated feasibility and consistent improvements in provider knowledge and attitudes. However, few studies examined whether these gains translated into sustained changes in clinical practice or system-level transformation. Most evaluations focused on short-term, self-reported outcomes, with limited use of validated tools or follow-up assessment. To advance the field, future research should extend beyond feasibility to evaluate the long-term effectiveness, sustainability and system-wide impact of TIC education across healthcare contexts.

### Patterns in outcome measurement

Across studies, outcomes clustered at the lower levels of Moore’s Expanded Outcomes Framework, primarily reflecting short-term learning rather than sustained behavioral or system-level change (Brown et al., 2021). Most evaluations focused on immediate post-training improvements in knowledge, attitudes or confidence, often assessed through pre–post self-report surveys. While these outcomes capture initial engagement and comprehension, they offer limited insight into how trauma-informed education translates into practice, relationships or patient outcomes over time. The use of validated measurement tools was inconsistent. Most studies relied on custom instruments tailored to specific training contexts, providing flexibility but limiting comparability across settings. A small subset used standardized tools such as the ARTIC-35 or ARTIC-45, indicating growing momentum toward structured evaluation (Baker et al., 2021). However, licensing requirements for these tools may limit accessibility and uptake, underscoring the need for freely available, validated measures aligned with trauma-informed principles.

Common methodological limitations also emerged. Many studies used small, voluntary samples, lacked control groups and measured outcomes immediately after training. Reliance on self-report data further limits objectivity, reflecting a feasibility-focused stage of research that constrains understanding of longer-term effects (Weiss et al., 2017). Future studies incorporating longitudinal follow-up and behavioral observation could better capture whether training influences provider practice, organizational culture and patient outcomes (Cerny et al., 2023).

### Gaps in evaluation and evidence

While training evaluations consistently demonstrate feasibility and short-term gains, there remains little evidence on whether these improvements lead to sustained change in provider behavior, patient outcomes or healthcare systems. The dominance of self-reported, proximal outcomes reflects a broader challenge in educational research: measuring what is easiest to assess rather than what matters most for implementation and equity (Purtle, 2020). Few studies employed longitudinal designs, observational methods or multi-level frameworks capable of capturing practice change over time. However, the appropriateness of outcome measures should also be considered in relation to the aims, intensity, duration and organizational reach of individual TIC educational interventions. The limited use of longitudinal and multi-level evaluation approaches has practical implications. Without evidence of impact beyond individual learning, the case for institutional investment in TIC education remains limited. Inconsistent measurement also hinders synthesis across studies, making it difficult to build a cumulative understanding of what constitutes effective TIC training (Simons et al., 2024). In addition, the aims and scope of TIC educational interventions varied considerably across studies, which may influence the selection and appropriateness of outcome measures used for evaluation. The absence of shared metrics parallels early stages in other health-education fields, where progress toward validated outcome frameworks was essential to advancing both research quality and policy relevance (Moore et al., 2009).

Another limitation concerns representativeness and reporting. Fewer than two-thirds of studies provided participant demographic information, and only a small subset distinguished between sex and gender (Stokes et al., 2024). Among those that did, samples were predominantly female, reflecting the gender distribution of certain caring professions but raising questions about the reach of TIC education across the wider healthcare workforce. Broader engagement across professions and genders will be essential to move TIC from a relational ideal associated with specific disciplines to a system-wide standard of care.

### Emerging relational and equity-oriented outcomes

A small but growing body of research is beginning to expand the scope of evaluation by operationalizing relational and equity-centered constructs as measurable outcomes. This marks an important shift from viewing TIC training primarily as an educational exercise to recognizing it as a mechanism for changing interpersonal, cultural and structural dimensions of care (Waite and Nardi, 2024). Several studies directly measured constructs such as empathy, bias awareness, cultural humility or provider empowerment, often through mixed methods designs that combined surveys with open-ended reflections or focus groups. Together, these studies illustrate how affective and relational change may represent early indicators of trauma-informed transformation within clinical teams (Dublin et al., 2021; Cerny et al., 2023). Despite these promising developments, such outcomes remain rarely included and inconsistently defined. Advancing this area will require clear conceptual models and validated instruments that capture empathy, cultural responsiveness and relational safety alongside traditional knowledge and competence outcomes, providing a more comprehensive understanding of how TIC education supports personal and systemic change.

### Implications for research and practice

Embedding outcome measurement within implementation frameworks can link educational change to clinical performance, patient experience and system transformation. Future evaluations should prioritize standardized, validated tools that capture both cognitive and relational domains of learning. Freely available instruments would enable comparability across studies and promote equity by reducing barriers related to licensing or cost. Mixed-methods and longitudinal designs are particularly suited to this field, allowing for both quantifiable assessment and exploration of how training influences culture, communication and safety within healthcare teams. From a practice perspective, integrating evaluation into routine education and quality-improvement systems will be critical for sustainability. Measuring not only what providers know, but how they enact TIC values, collaboration, empowerment and equity, can help organizations understand where implementation is effective and where structural supports are needed. Ultimately, advancing outcome measurement in TIC education is not only a methodological challenge but also an ethical imperative: without evidence of impact, trauma-informed approaches risk being endorsed in principle but underrealized in practice.

### Strengths and limitations

A key strength of this review lies in its dual analytic framing. By mapping outcomes within Moore’s Expanded Outcomes Framework while also identifying relational and equity-informed dimensions, the review extends conventional approaches to evaluating healthcare education. This allowed for a more comprehensive synthesis that captured both measurable learning outcomes and emerging constructs reflecting the values of trauma-informed practice. The review also benefited from a rigorous and transparent process, including librarian-supported search strategy development, dual independent screening and structured data extraction. Together, these methods enhanced reliability and replicability.

However, several limitations should be noted. The exclusion of gray literature and non-English publications may have resulted in the omission of relevant studies, particularly from community-based or low- and middle-income settings where trauma-informed approaches are often developed outside of peer-reviewed channels. Additionally, the inclusion of “ARTIC” as a search term – based on its prominence in early exploratory searches – may have contributed to overrepresentation of studies using that tool. Finally, the diversity of study designs and outcome measures limited the ability to compare findings quantitatively, underscoring the need for greater standardization in future research. This review included studies from multiple countries, and as noted, the majority were conducted in the United States, reflecting the current concentration of TIC implementation research within North American healthcare systems. The use of additional regional databases may have identified further studies from non-US settings, and the current evidence base may therefore underrepresent TIC educational approaches from other healthcare systems and sociocultural contexts. We understand that across regions, healthcare contexts differ substantially with respect to workforce roles, organizational structures, access to training resources and models of care delivery. These contextual differences may influence how TIC educational interventions are designed, implemented and evaluated. As a result, outcome measures that are feasible or prioritized in one healthcare system may not be directly transferable to another.

### Future directions

Future research should prioritize the development and use of outcome measures that are freely accessible, psychometrically robust and applicable across diverse care settings and populations. Standardized tools would enable comparison across studies and support the cumulative advancement of evidence in TIC education. At the same time, evaluation frameworks should remain flexible enough to accommodate differences in intervention aims, intensity, setting and intended outcomes across TIC educational initiatives. Equally important is the inclusion of outcomes that extend beyond immediate learning to capture long-term, system-level change.

Methodologically, longitudinal and mixed-methods designs are well suited to this work, allowing researchers to examine both sustained behavioral change and the contextual factors that influence implementation. Future evaluations should also integrate relational and equity-informed metrics, such as empathy, cultural humility and relational safety, to reflect the full scope of TIC values. Collaborative research partnerships between healthcare organizations, educators and communities can accelerate progress by ensuring that outcome measures are not only scientifically rigorous but also meaningful, feasible and aligned with the lived realities of practice. In conclusion, this review demonstrates that while TIC education is advancing rapidly across healthcare systems, evaluation practices remain methodologically fragmented. Establishing standardized, equity-informed measures will be essential for ensuring that TIC training contributes to sustainable, system-level change.

## Supporting information

10.1017/gmh.2026.10277.sm001 Vishwanath et al. supplementary material Vishwanath et al. supplementary material

## Data Availability

Data available from the corresponding author on request.

## Supplementary material

The supplementary material for this article can be found at http://doi.org/10.1017/gmh.2026.10277.

## Long descriptions

### Figure 1. Long description

The flowchart is divided into three vertical sections.

1. Identification Phase at the top.

Left box: Studies from databases/registers (n = 3603) including Embase (n = 974), C I N A H L (n = 940), PsycINFO (n = 858), M E D L I N E (n = 680), and E R I C (n = 151).

Right box: References from other sources (n = 0) including citation searching and grey literature.

An arrow leads to a side box: References removed (n = 1253) due to manual duplicates (n = 2) and Covidence duplicates (n = 1251).

2. Screening Phase in the middle.

Studies screened (n = 2350) leads to Studies excluded (n = 2270).

Studies sought for retrieval (n = 80) leads to Studies not retrieved (n = 0).

Studies assessed for eligibility (n = 80) leads to Studies excluded (n = 52). Reasons for exclusion include wrong setting (n = 5), wrong population (n = 15), not peer-reviewed (n = 5), wrong study design (n = 20), no relevant outcomes measured (n = 2), and no T I C educational intervention (n = 5).

3. Included Phase at the bottom.

A final box indicates Studies included in review (n = 28).

Navigate back to Figure 1..

### Table 1. Long description

The table consists of 8 columns: Author and year of publication, Country, Study design, Setting, Participants, Duration of intervention, Intervention, and Primary measure.

Key entries include:

* Raja et al. 2015 (USA): Program evaluation in a dental school with second-year students; two 3.5-hour sessions involving lectures and role-play.

* Brown et al. 2021 (USA): Quasi-experimental study in a medical school; 3.5-hour in-person session with didactics and role-plays for medical and dental students.

* Niimura et al. 2019 (Japan): Quasi-experimental study in psychiatric hospitals; 4.5-hour in-person lecture using A R T I C-45 and A R T I C-35 measures.

* Ford et al. 2021 (USA): Quality improvement in N I C U settings over 2.5 years using hybrid meetings.

* Cerny et al. 2023 (USA): Mixed methods research in a continuing education program; 6-week self-study and 4-hour in-person session using A R T I C-35.

* Stewart et al. 2024 (Canada): Mixed methods research in community agencies; 12-hour hybrid intervention measured by A R T I C-45.

* Kopstick et al. 2025 (USA): Quasi-experimental study at an academic medical conference; 90-minute in-person workshop.

* Lamberson et al. 2025 (USA): Quasi-experimental study in an academic emergency department; 4-hour in-person session for E D staff and E M T s.

Common primary measures across studies include custom Likert scale surveys, the Attitudes Related to Trauma-Informed Care (A R T I C) scales, and qualitative interviews.

Navigate back to Table 1..

### Figure 2. Long description

The y-axis is labeled Number of Studies with a scale from 0 to 30 in increments of 5. The x-axis lists eight categories from Level 1 to Level 7. A legend at the bottom identifies dark blue as Direct Measurement and light blue as Indirect Measurement.

* Level 1 Participation: 29 studies, all Direct Measurement.

* Level 2 Satisfaction: 23 studies total, with 22 Direct and 1 Indirect.

* Level 3 A Declarative knowledge: 25 studies total, with 18 Direct and 7 Indirect.

* Level 3 B Procedural Knowledge: 26 studies total, with 6 Direct and 20 Indirect.

* Level 4 Competence: 24 studies total, with 8 Direct and 16 Indirect.

* Level 5 Performance: 14 studies, all Direct Measurement.

* Level 6 Patient Health: 0 studies recorded.

* Level 7 Community Health: 6 studies, all Indirect Measurement.

Navigate back to Figure 2..
