# Supplementary material for: Outcome measurement in trauma-informed care education across healthcare settings: A scoping review
Source: Glob Ment Health (Camb). 2026 Jul 23;13:e164. doi: 10.1017/gmh.2026.10277 (PMC13433329; doi:10.1017/gmh.2026.10277)
Supplement: Supplementary file 1 [file S2054425126102775sup001.pdf]

Search Strategy

HTHSCI 4C09

Databases Searched: MEDLINE, Embase, APA PsycINFO, CINAHL, ERIC
Total Returned (subtracting duplicates): 2352

MEDLINE

Database(s): OVID Medline Epub Ahead of Print, In-Process & Other Non-Indexed Citations, Ovid MEDLINE(R) Daily and Ovid MEDLINE(R) 1946 to Present
Search Strategy:

#	Searches	Results
1	(trauma informed care or trauma informed practice or trauma informed approach or trauma integrated care or trauma centered care or trauma sensitive care or trauma sensitive practice or trauma sensitive approach or trauma responsive care or trauma responsive practice or trauma responsive approach).mp.	1848
2	(education or course* or workshop* or train* or teach* or curricul* or program*).mp.	3482390
3	((knowledge or skill* or attitude*) adj3 (measure* or assess*)).mp.	58666
4	(outcome measure* or outcome assess*).mp.	394747
5	("attitudes related to trauma informed care" or "ARCTIC scale" or "secondary traumatic stress" or "STS scale").mp.	969
6	exp "Attitude of Health Personnel"/	174415
7	exp education, professional/ or exp educational measurement/ or exp professional competence/	438318
8	exp Staff Development/	10012
9	2 or 3 or 4 or 5 or 6 or 7 or 8	3944786
10	(doctor* or physician* or clinician* or surgeon* or nurs* or dentist* or pharmac* or psycholog* or psychiatr* or occupational therap* or physical therap* or physiotherap* or speech language patholog* or radiolog* or anesth* or pediatric* or cardiolog* or oncolog* or dermatolog* or endocrinolog* or gastroenterolog* or neurolog* or urolog* or ophthalmolog* or rheumatolog* or nephrolog* or optometr* or orthopedic* or therapist* or audiologist* or medical laboratory personnel* or obstetric* or anatomist* or public health prof* or genetic counsel* or midwife* or doula* or paramedic* or medical assistant* or physician assistant* or neonatolog* or hematolog* or prosthetist* or geriatric* or social worker* or medical student* or health administrat* or care specialist* or healthcare pro* or health personnel* or healthcare work*).mp.	10696518
11	exp Health Personnel/	644068
12	10 or 11	10766110
13	1 and 9 and 12	680

Results Returned: 680

APA PsycINFO

Database(s): **APA PsycInfo** 1806 to October 2024 Week 3

Search Strategy:

#	Searches	Results
1	(trauma informed care or trauma informed practice or trauma informed approach or trauma integrated care or trauma centered care or trauma sensitive care or trauma sensitive practice or trauma sensitive approach or trauma responsive care or trauma responsive practice or trauma responsive approach).mp.	2587
2	exp trauma-informed care/	1585
3	1 or 2	2797
4	(education or course* or workshop* or train* or teach* or curricul* or program*).mp.	1512266
5	((knowledge or skill* or attitude*) adj3 (measure* or assess*)).mp.	51102
6	(outcome measure* or outcome assess*).mp.	60424
7	("attitudes related to trauma informed care" or "ARCTIC scale" or "secondary traumatic stress" or "STS scale").mp.	1491
8	exp health personnel attitudes/	33767
9	exp professional competence/ or exp professional development/	77507
10	exp educational measurement/ or exp educational objectives/	25750
11	4 or 5 or 6 or 7 or 8 or 9 or 10	1634082
12	(doctor* or physician* or clinician* or surgeon* or nurs* or dentist* or pharmac* or psycholog* or psychiatr* or occupational therap* or physical therap* or physiotherap* or speech language patholog* or radiolog* or anesth* or pediatric* or cardiolog* or oncolog* or dermatolog* or endocrinolog* or gastroenterolog* or neurolog* or urolog* or ophthalmolog* or rheumatolog* or nephrolog* or optometr* or orthopedic* or therapist* or audiologist* or medical laboratory personnel* or obstetric* or anatomist* or public health prof* or genetic counsel* or midwife* or doula* or paramedic* or medical assistant* or physician assistant* or neonatolog* or hematolog* or prosthetist* or geriatric* or social worker* or medical student* or health administrat* or care specialist* or healthcare pro* or health personnel* or healthcare work*).mp.	1943010
13	exp health personnel/	206081
14	12 or 13	1974287
15	3 and 11 and 14	858

Results Returned: 858

Embase

Database(s): **Embase** 1974 to 2024 October 22

Search Strategy:

#	Searches	Results
1	(trauma informed care or trauma informed practice or trauma informed approach or trauma integrated care or trauma centered care or trauma sensitive care or trauma sensitive practice or trauma sensitive approach or trauma responsive care or trauma responsive practice or trauma responsive approach).mp.	2263
2	(education or course* or workshop* or train* or teach* or curricul* or program*).mp.	5261414
3	((knowledge or skill* or attitude*) adj3 (measure* or assess*)).mp.	82883
4	(outcome measure* or outcome assess*).mp.	1201673
5	("attitudes related to trauma informed care" or "ARCTIC scale" or "secondary traumatic stress" or "STS scale").mp.	1126
6	exp health personnel attitude/ or exp professional competence/ or exp professional development/ or exp staff training/ or exp allied health education/ or exp nursing education/ or exp research based nursing education/ or exp graduate education/ or exp medical education/ or exp postgraduate education/ or exp clinical education/ or exp masters education/ or exp rescue personnel attitude/ or exp dental health education/ or exp podiatry education/ or exp occupational therapy education/ or exp paramedical education/ or exp midwifery education/ or exp pharmacy education/ or exp physician assistant education/ or exp continuing education provider/ or exp interdisciplinary education/ or exp interprofessional education/ or exp "outcome of education"/ or exp outcome assessment/ or exp education program/ or exp social work education/ or exp continuing education/	1690927
7	2 or 3 or 4 or 5 or 6	6397115
8	(doctor* or physician* or clinician* or surgeon* or nurs* or dentist* or pharmac* or psycholog* or psychiatr* or occupational therap* or physical therap* or physiotherap* or speech language patholog* or radiolog* or anesth* or pediatric* or cardiolog* or oncolog* or dermatolog* or endocrinolog* or gastroenterolog* or neurolog* or urolog* or ophthalmolog* or rheumatolog* or nephrolog* or optometr* or orthopedic* or therapist* or audiologist* or medical laboratory personnel* or obstetric* or anatomist* or public health prof* or genetic counsel* or midwife* or doula* or paramedic* or medical assistant* or physician assistant* or neonatolog* or hematolog* or prosthetist* or geriatric* or social worker* or medical student* or health administrat* or care specialist* or healthcare pro* or health personnel* or healthcare work*).mp.	12408999
9	exp health care personnel/	2119072
10	8 or 9	12723097
11	1 and 7 and 10	974

Results Returned: 974

CINAHL

EBSCOhost

Wed, October 23, 2024 10:01:33 AM

#	Query	Limiters/Expanders	Last Run Via	Results
S15	S1 AND S10 AND S14	Search modes - Proximity	Interface - EBSCOhost Research Databases<br>Search Screen - Advanced Search<br>Database - CINAHL	940
S14	S11 OR S12 OR S13	Search modes - Proximity	Interface - EBSCOhost Research Databases<br>Search Screen - Advanced Search<br>Database - CINAHL	5,982,063
S13	(MH "Health Labor Supply+")	Search modes - Proximity	Interface - EBSCOhost Research Databases<br>Search Screen - Advanced Search<br>Database - CINAHL	673,739
S12	(MH "Health Personnel+")	Search modes - Proximity	Interface - EBSCOhost Research Databases<br>Search Screen - Advanced Search<br>Database - CINAHL	663,930
S11	TX ((doctor* or physician* or clinician* or surgeon* or nurs* or dentist* or pharmac* or psycholog* or psychiatr* or occupational therap* or physical therap* or physiotherap* or speech language patholog* or radiolog* or anesth* or pediatric* or cardiolog* or oncolog* or dermatolog* or endocrinolog* or gastroenterolog* or neurolog* or urolog* or ophthalmolog* or rheumatolog* or nephrolog* or optometr* or orthopedic* or therapist* or audiologist* or medical laboratory personnel* or obstetric* or anatomist* or public health prof* or genetic counsel* or midwife* or doula* or paramedic* or medical assistant* or physician assistant* or neonatolog* or hematolog* or prosthetist* or geriatric* or social worker* or medical student* or health administrat* or care specialist* or healthcare pro* or health personnel* or healthcare work*))	Search modes - Proximity	Interface - EBSCOhost Research Databases<br>Search Screen - Advanced Search<br>Database - CINAHL	5,902,776
S10	S2 OR S3 OR S4 OR S5 OR S6 OR S7 OR S8 OR S9	Search modes - Proximity	Interface - EBSCOhost Research Databases<br>Search Screen - Advanced Search<br>Database - CINAHL	2,332,630
S9	(MH "Professional Competence+") OR (MH "Clinical Competence+") OR (MH "Competency Assessment")	Search modes - Proximity	Interface - EBSCOhost Research Databases<br>Search Screen - Advanced Search<br>Database - CINAHL	95,280
S8	(MH "Educational Measurement+") OR (MH "Education, Competency-Based") OR (MH "Education, Health Sciences+") OR (MH "Education, Clinical+") OR (MH "Staff Development+")	Search modes - Proximity	Interface - EBSCOhost Research Databases<br>Search Screen - Advanced Search<br>Database - CINAHL	423,202
S7	(MH "Attitude Measures") OR (MH "Psychotherapist Attitudes") OR (MH "Dentist Attitudes") OR (MH "Pharmacist Attitudes") OR (MH "Employee Attitudes") OR (MH "Audiologist Attitudes") OR (MH "Physician Attitudes") OR (MH "Midwife Attitudes") OR (MH "Nurse Attitudes") OR (MH "Faculty Attitudes") OR (MH "Dental Hygienist Attitudes") OR (MH "Social Worker Attitudes") OR (MH "Respiratory Therapist Attitudes") OR (MH "Physician Assistant Attitudes") OR (MH "Physical Therapist Attitudes") OR (MH "Occupational Therapist Attitudes") OR (MH "Emergency Medical Technician Attitudes") OR (MH "Speech-Language Pathologist Attitudes") OR (MH "Attitude of Health Personnel+")	Search modes - Proximity	Interface - EBSCOhost Research Databases<br>Search Screen - Advanced Search<br>Database - CINAHL	156,098
S6	(MH "Professional Development+")	Search modes - Proximity	Interface - EBSCOhost Research Databases<br>Search Screen - Advanced Search<br>Database - CINAHL	43,998
S5	TX (("attitudes related to trauma informed care" or "ARCTIC scale" or "secondary traumatic stress" or "STS scale"))	Search modes - Proximity	Interface - EBSCOhost Research Databases<br>Search Screen - Advanced Search<br>Database - CINAHL	836
S4	TX ((outcome measure* or outcome assess*))	Search modes - Proximity	Interface - EBSCOhost Research Databases<br>Search Screen - Advanced Search<br>Database - CINAHL	167,752
S3	TX (((knowledge or skill* or attitude*) w3 (measure* or assess*))	Search modes - Proximity	Interface - EBSCOhost Research Databases<br>Search Screen - Advanced Search<br>Database - CINAHL	26,795
S2	TX ((education or course* or workshop* or train* or teach* or curricul* or program*))	Search modes - Proximity	Interface - EBSCOhost Research Databases<br>Search Screen - Advanced Search<br>Database - CINAHL	2,083,046
S1	TX ((trauma informed care or trauma informed practice or trauma informed approach or trauma integrated care or trauma centered care or trauma sensitive care or trauma sensitive practice or trauma sensitive approach or trauma responsive care or trauma responsive practice or trauma responsive approach))	Search modes - Proximity	Interface - EBSCOhost Research Databases<br>Search Screen - Advanced Search<br>Database - CINAHL	1,708

Results Returned: 940

ERIC

(trauma informed care OR trauma informed practice OR trauma informed approach OR trauma integrated care OR trauma centered care OR trauma sensitive care OR trauma sensitive practice OR trauma sensitive approach OR trauma responsive care OR trauma responsive practice OR trauma responsive approach) AND (education OR course OR workshop OR train OR teach OR curricula OR program OR outcome measurement OR outcome assessment OR knowledge assessment OR attitude assessment OR skill assessment OR "attitudes related to trauma informed care" OR "ARCTIC scale" OR "secondary traumatic stress" OR "STS scale") AND (doctor OR physician OR clinician OR surgeon OR nurse OR dentist OR pharmacist OR psychologist OR psychiatrist OR occupational therapist OR physical therapist OR physiotherapist OR speech language pathologist OR radiologist OR anesthesiologist OR pediatrician OR cardiologist OR oncologist OR dermatologist OR endocrinologist OR gastroenterologist OR neurologist OR urologist OR ophthalmologist OR rheumatologist OR nephrologist OR optometrist OR orthopedic OR therapist OR audiologist OR medical laboratory personnel OR obstetrician OR anatomist OR public health professional OR genetic counsellor OR midwife OR doula OR paramedic OR medical assistant OR physician assistant OR neonatologist OR hematologist OR prosthetist OR geriatrician OR social worker OR medical student OR health administrator OR care specialist OR healthcare professional OR health personnel OR healthcare worker)

Results Returned: 151
