# Supplementary material for: Outcome measurement in trauma-informed care education across healthcare settings: A scoping review
Source: Glob Ment Health (Camb). 2026 Jul 23;13:e164. doi: 10.1017/gmh.2026.10277 (PMC13433329; doi:10.1017/gmh.2026.10277)
Supplement: Peer review history

# Reviewer report: Review: Outcome measurement in trauma-informed care education across healthcare settings: A scoping review — R0/PR2

**DOI:** 10.1017/gmh.2026.10277.pr2

**Authors:** Nicole Maiorano

**Affiliations:** Centre for Global Health, Trinity College Dublin, Ireland

Thank you for sharing this review. As the authors describe, it is an important time to understand evaluations of TIC in healthcare settings due to the growth and investment we’ve seen in this approach. Please note, the recommendation of major revisions was provided based on the comments related to expanding the analysis. I hope the feedback provided below can support the review provided.

Introduction

• The acronym “TIC” should be written out in full (i.e., “trauma-informed care”) before its first use in the Introduction (page 4, line 49)

• Throughout the Introduction references to healthcare specifically and organisations broadly are made which may cause confusion for readers. Specifically, when TIC is first introduced it is done using healthcare related terms (i.e., “patients and providers”, page 4, line 50). It may help to describe the use of TIC broadly and then specifically in relation to healthcare more clearly to help a reader understand the scope of TIC and of this review.

Methods

• There is a formatting error in the arrows and lines of the PRISMA chart (page 9). Additionally, the box for ongoing studies can be removed.

• Can more information be provided on the definition of ‘TIC’ used for the review’s screening and inclusion criteria? For example, the search criteria include a wide range of related terms. Were studies included if they used TIC related language to describe their training (e.g., trauma-aware)?

Results

• References for the studies included in the review are missing from the reference list. Please review and update.

• Based on the criteria that articles were included if they provided training to healthcare professionals, the inclusion of a publication that provided training to childcare professionals in a community and social services setting (Diercks et al., 2023) may not be correct. Can this please be reviewed?

• The authors should consider expanding the analysis to include the aims of the training interventions. A main point of the Discussion is that the training evaluation measures cannot capture wider organisational changes. However, the evaluation measures should be determined based on the intervention’s aims and it is therefore challenging to make conclusions about the measures without an understanding of the intervention aims.

Discussion

• Similarly to the above point, it is challenging to know if the evaluation measures are appropriate for the training aims and targets. It may not be appropriate to anticipate system-level transformation following a short training with a portion of a healthcare organisation’s staff. Further exploration of this point and incorporation of relevant literature regarding what should be measured and how it can be measured may strengthen the Discussion.

• The point about inconsistent measurements can be explored in more depth. As there may be variations in the aims, nature, and predicted outcomes of TIC trainings based on their context and design, can we anticipate the same measures being used? If so, what could this look like and what does previous research suggest for this?

# Reviewer report: Review: Outcome measurement in trauma-informed care education across healthcare settings: A scoping review — R0/PR3

**DOI:** 10.1017/gmh.2026.10277.pr3

**Authors:** Lene Lauge Berring

Thank you for the opportunity to review Outcome Measurement in Trauma Informed Care Education Across Healthcare Settings: A Scoping Review.

Overall, the review is clearly written and addresses an important and timely topic. In relation to the global review criteria, there are, however, several areas where the manuscript could be further strengthened.

Coverage of global content

The review draws on international literature, but the global dimension could be articulated more explicitly. It is somewhat difficult to determine how consistently the PCC framework (Population, Concept, Context) has been applied, particularly regarding the context element. A clearer explanation of how healthcare settings differ across world regions—and how these differences influence TIC educational interventions—would enhance the global relevance of the review.

Aim versus outcomes

There appears to be a slight conceptual mismatch between the stated aim, which focuses on educational interventions, and the outcome measures identified, which seem more closely related to implementation processes. It may be helpful for the authors to revisit this alignment to ensure conceptual coherence across diverse global contexts.

Use of evidence and best practice

The manuscript convincingly highlights the lack of systematic synthesis regarding outcome measurement in TIC education. The reference to recent curricular analyses (e.g., Gerber et al., 2024) is appropriate, though the global implications of these gaps could be expanded. For instance, differences in TIC adoption, training standards, and evaluation practices across regions could be discussed more explicitly.

Representation of global perspectives

Given that the journal prioritises a global perspective, the review would benefit from a clearer description of the geographical distribution of included studies. At present, it is not entirely clear whether the evidence base is dominated by particular countries or whether a broad range of healthcare systems is represented. This makes it difficult to assess the global applicability of the findings.

Strengths

The use of Moore’s Expanded Outcomes Framework is a notable strength. It provides a structured and comparable way to analyse diverse studies and is well justified as an evaluative model for educational interventions across settings.

Recommendations for improvement

• Clarify how the PCC framework guided study selection, particularly with regard to global context.

• Strengthen the alignment between the stated aim (education) and the identified outcomes (implementation).

• Provide a clearer mapping of the geographical distribution of included studies.

• Expand the discussion to address how global variation in healthcare systems shapes TIC education and outcome measurement.

• Consider integrating perspectives from TIC practitioners or educators to contextualise findings across regions.

Finally, it appears that different typographical styles are used in parts of the manuscript (e.g., p. 6/31), and harmonising these would improve readability.

# Reviewer report: Review: Outcome measurement in trauma-informed care education across healthcare settings: A scoping review — R0/PR4

**DOI:** 10.1017/gmh.2026.10277.pr4

**Authors:** Amy Weil

This is an excellent and much needed review of the next stage in integrating TIC into medical education - defining and standardizing the outcomes we are looking for so that improvement can be measured and progress can be made. Bravo! I hope to integrate your excellent suggestions regarding metrics to use and that formulation of the levels and agree wholeheartedly that a way to measure the equity aspects is very much needed.

# Reviewer report: Review: Outcome measurement in trauma-informed care education across healthcare settings: A scoping review — R0/PR5

**DOI:** 10.1017/gmh.2026.10277.pr5

Teaching trauma informed health care is rapidly growing in interest, uptake, inclusion in curricula, health system attention, and research. The world has moved past “do we need trauma informed care?” to “how do we do it and how do we teach it?” Teaching TIC is still in its infancy and evaluation methods are either absent or inadequate. This global review of the state of outcome measurement in TIC education is very needed. The authors have done a good job of addressing this and the addition of Moore’s Expanded Outcomes Framework adds to recent articles on this topic. The authors have succeeded in showing that much more work needs to be done – particularly around standardization of evaluation of training and the need to extend outcomes to capture long-term, system level change.

There are some key issues that need to be addressed:

1. This is meant to be a global review, yet 24 of 29 studies are from the USA. This certainly may be the state of the literature currently, but I would like to know what efforts were made to find non-USA-based studies. Are there other international databases that could have been searched that were left out? If so, why? I would also recommend adding this issue to potential limitations.

2. The only study included that was not from an English-speaking country was Dierickx, et al. and only included childcare professional caregivers as participants. This participant group does not meet your eligibility criteria for studies involving healthcare providers. Why was it included? Also, please explain how this study made it into your study inclusion, as it does not appear to meet your criteria.

3. Your retrieval ends October 2024, which is now about 18 months ago. Did you consider running your search again to see if any key articles may have been missed? This field is changing so fast. I feel your review would be stronger if it is as up to date as possible.

a. I am aware of a few articles that likely would have been included that have been published since October 2024.

i. A communication skills in TIC evaluation form has been developed and used by Thang and colleagues. (1&2)

1. Thang CK, Kucaj S, Arellano F, Lay K, Wilhalme H, Szilagyi M. Pediatric Approach to Trauma, Treatment, and Resilience (PATTeR) for Trainees: A Standardized Trauma-Informed Care Training for Pediatric Residents. MedEdPORTAL. 2026 Mar 20;22:11584. doi: 10.15766/mep_2374-8265.11584. PMID: 41869457

2. Thang CK, Kucaj S, Arellano F, Lay K, Wilhalme H, Szilagyi M. Pediatric Approach to Trauma, Treatment, and Resilience (PATTeR) for Trainees: A Standardized Trauma-Informed Care Training for Pediatric Residents. MedEdPORTAL. 2026 Mar 20;22:11584. doi: 10.15766/mep_2374-8265.11584. PMID: 41869457

3. Nunziato J, Lessard C, Karp N, Lane H, Locklear T, Simcox K. A Community-Based Trauma-Informed Care Curriculum on Women’s Health for Third-Year Medical Students. MedEdPORTAL. 2026 Feb 18;22:11578. doi: 10.15766/mep_2374-8265.11578. PMID: 41716725

4. Chin J, Sheth M, Britz S, Owens L, Zhang Y. A Trauma-informed Care Curriculum for Perinatal Providers, Staff, and Learners. MedEdPORTAL. 2025 Dec 9;21:11563. doi: 10.15766/mep_2374-8265.11563. PMID: 41378372

4. The first reference needs correction. This is the citation for the first use of the SAMHSA TIC principles in 2014. This can be hard to cite currently, as the original citation has been removed. I would recommend: https://www.samhsa.gov/mental-health/trauma-violence/trauma-informed-approaches-programs as this has the principles listed and SAMHSA is the original creator of these principles.

# Editor report: Recommendation: Outcome measurement in trauma-informed care education across healthcare settings: A scoping review — R0/PR6

**DOI:** 10.1017/gmh.2026.10277.pr6

**Affiliations:** Department of Global Health and Social Medicine, Harvard Medical School, United States

No accompanying comment.

# Editor report: Decision: Outcome measurement in trauma-informed care education across healthcare settings: A scoping review — R0/PR7

**DOI:** 10.1017/gmh.2026.10277.pr7

**Affiliations:** Johns Hopkins University Bloomberg School of Public Health, United States

No accompanying comment.

# Reviewer report: Review: Outcome measurement in trauma-informed care education across healthcare settings: A scoping review — R1/PR9

**DOI:** 10.1017/gmh.2026.10277.pr9

**Authors:** Nicole Maiorano

**Affiliations:** Centre for Global Health, Trinity College Dublin, Ireland

Thank you for the opportunity to review this article. I believe the feedback was well incorporated and I am satisfied with the revisions made.

One small note, as the number of included articles was updated (from 29 to 28), the authors may need to check and confirm some of the numbers reported in the results section (e.g., page 13, line 295, the number and percentage may need to be updated; line 306 the percentage may need to be updated). Additionally, the number of included studies needs to be updated on page 17, line 417.

# Reviewer report: Review: Outcome measurement in trauma-informed care education across healthcare settings: A scoping review — R1/PR10

**DOI:** 10.1017/gmh.2026.10277.pr10

Thank your for you attention to the suggested edits.

I remained confused about the timeline of the search and included studies. You say that the search was through October 2024, yet you have included studies in your review from 2025? Please explain how this occurred and correct the error.

My major issue with the paper is lack of timeliness in this rapidly changing world of TIC education. You mention that rerunning the search is beyond the scope of your review, but scoping reviews often add additional, late-breaking studies, that fit inclusion criteria, after main search is done - so I don’t find this an adequate explanation for not including the most current literature. And it appears that you have included studies that were published after your review was done so this argument doesn’t make sense.

If you reviewed papers from after October 2024, then please state that, how it was done, what the actual search date was.

Since it is obvious that there will be missing articles from early 2026 that are not included, that should be explicitly stated and I don’t see that in your limitations.

# Reviewer report: Review: Outcome measurement in trauma-informed care education across healthcare settings: A scoping review — R1/PR11

**DOI:** 10.1017/gmh.2026.10277.pr11

**Authors:** Lene Lauge Berring

Thank you for allowing me to revisit this important review. It is evident that the authors have made a sincere effort to revise the manuscript based on the reviewers’ feedback. However, I still have a few questions, particularly given that the Joanna Briggs Institute methodology (Tricco et al.) was used.

JBI recommends that the key PCC elements (Population, Concept, Context) are made explicitly visible throughout the review. The authors refer to an explanation on line 206, but I am unfortunately unable to see how PCC has actually guided the formulation of the research question or the search strategy. Clarifying this connection would significantly strengthen the manuscript.

Furthermore, Joanna Briggs emphasizes the involvement of knowledge users in the review process. I cannot see evidence that such users were engaged here, and it would be helpful either to describe how they were involved or to justify their absence.

Thank you again for the opportunity to re‑read this substantial piece of work.

# Editor report: Recommendation: Outcome measurement in trauma-informed care education across healthcare settings: A scoping review — R1/PR12

**DOI:** 10.1017/gmh.2026.10277.pr12

**Affiliations:** Department of Global Health and Social Medicine, Harvard Medical School, United States

No accompanying comment.

# Editor report: Decision: Outcome measurement in trauma-informed care education across healthcare settings: A scoping review — R1/PR13

**DOI:** 10.1017/gmh.2026.10277.pr13

**Affiliations:** Johns Hopkins University Bloomberg School of Public Health, United States

No accompanying comment.

# Editor report: Recommendation: Outcome measurement in trauma-informed care education across healthcare settings: A scoping review — R2/PR15

**DOI:** 10.1017/gmh.2026.10277.pr15

**Affiliations:** Department of Global Health and Social Medicine, Harvard Medical School, United States

No accompanying comment.

# Editor report: Decision: Outcome measurement in trauma-informed care education across healthcare settings: A scoping review — R2/PR16

**DOI:** 10.1017/gmh.2026.10277.pr16

**Affiliations:** Johns Hopkins University Bloomberg School of Public Health, United States

No accompanying comment.
